# Coagulation profile in hospitalized children with COVID-19: pediatric age dependency and its impact on long COVID development

**DOI:** 10.3389/fimmu.2024.1363410

**Published:** 2024-03-06

**Authors:** Oksana Boyarchuk, Vita Perestiuk, Tetyana Kosovska, Liubov Volianska

**Affiliations:** Department of Children’s Diseases and Pediatric Surgery, I. Horbachevsky Ternopil National Medical University, Ternopil, Ukraine

**Keywords:** COVID-19, SARS-CoV-2 infection, long COVID, coagulation profile, children, risk factors

## Abstract

**Introduction:**

Pulmonary endotheliopathy and microvascular immunothrombosis play a key role in acute COVID-19. Moreover, persistent endotheliopathy and heightened coagulability frequently occur in individuals recovering from COVID-19, suggesting the intriguing possibility of their role in the development of long COVID. The aim of our study was to investigate the coagulation profile in patients with COVID-19 based on age and their role in the development of long COVID.

**Methods:**

We conducted a prospective single-center cohort study from September 2022 to August 2023. The study involved 190 patients younger than 18 years who were hospitalized at the Ternopil City Children's Hospital, Ukraine due to COVID-19. Patients underwent determination of coagulation profile in addition to the general clinical examination. After discharge from the hospital, patients were monitored for the presence of long COVID symptoms. Among the 157 participants who consented for follow-up, 62 patients (39.5%) had long COVID symptoms according to the WHO definition, while the rest (95 patients) did not have symptoms of long COVID (fully recovered).

**Results:**

The study revealed the normal count of platelets in the majority of patients (86.8%), whereas abnormalities in the coagulation profile were revealed in 94.5% of children with COVID-19, and these changes were age-dependent. The patients were mostly presented with increased activated partial thromboplastin time (69.1%), prothrombin time (PT) (39.8%) and D-dimer (45.0%). There was no significant difference between the median of platelet levels and coagulation profile indicators between the groups with long COVID and recovered. Among children who developed persistent long COVID symptoms there was a statistically higher percentage of abnormal PT values (53% versus 36.1%, p=0.0432), with no significant differences in other coagulation profile indicators. Abnormal PT along with female gender, comorbidities, especially allergic pathology, nutritional disorder, including obesity, were determined as potential risk factors of the long COVID development (Odds ratio - 2.0611; 95% 1.0179-4.1737, p=0.0445).

**Conclusions:**

The study highlights the need for more extensive research into the coagulation profiles of pediatric populations, considering age-specific factors. This could enhance our understanding of thromboinflammation in COVID-19 and its potential contribution to the development of persistent symptoms.

## Introduction

COVID-19 is an acute infectious disease caused by the SARS-CoV-2 virus. Initially believed to primarily affect the respiratory system, causing interstitial pneumonia and acute respiratory distress syndrome, it has been later investigated that, besides lung disease, COVID-19 can lead to a broad spectrum of disorders involving various organs in pathological processes or complications directly or indirectly related to this infection (1). It is now established that pulmonary endotheliopathy and microvascular immunothrombosis play a key role in acute COVID-19 (2).

While COVID-19 is more severe and deadly in older individuals, data from the American Academy of Pediatrics (AAP) and the Association of Pediatricians indicate that children make up around 18% of all recorded COVID-19 cases in the United States. Among these cases, 3% require hospitalization, and the mortality rate is 0.2%. Nevertheless, among children admitted to hospitals, a significant percentage (21-40%) needed intensive care, and a notable proportion (6-9%) required invasive mechanical ventilation, with up to 3% experiencing fatal outcomes (1, 3–5). Those with pre-existing chronic conditions such as diabetes, obesity, congenital heart issues, chronic respiratory and neurological disorders, and immunodeficiency were more susceptible to severe cases, necessitating hospitalization (3, 6, 7).

At the onset of the pandemic, a propensity for thrombotic coagulopathy due to abnormal coagulation status became evident, posing a significant challenge for COVID-19 patients (8). This “COVID-19 coagulopathy” was associated with adverse outcomes in patients, including thromboembolism, stroke, and death (9–12). It was observed in patients with a severe course of COVID-19 and involved diffuse coagulation activation but with a pattern distinct from classical disseminated intravascular coagulation (DIC) syndrome. Although D-dimers may be noticeably elevated, in COVID-19, prothrombin time and activated partial thromboplastin time are only slightly prolonged, fibrinogen is increased, and the platelet count is usually normal or moderately decreased in a significant portion of hospitalized patients (13, 14). COVID-19 coagulopathy is prothrombotic and does not result in hemorrhagic complications (14).

The pathogenesis of “COVID-19 coagulopathy” is multifactorial, involving endothelial cell damage, inflammation, platelet activation, and changes in coagulation factors, collectively leading to “thromboinflammation.” It is unclear whether endothelial cell damage is directly related to viral infection or is a subsequent inflammatory process, partly mediated by angiotensin-converting enzyme. The primary receptor for SARS-CoV-2 is expressed at much higher levels in the respiratory tract’s epithelial cells than in endothelial cells (15). This leads to the release of cytokines and activation of interleukins, interferons, and tissue necrosis factors (16). Intense inflammatory signals damage the protective barrier of endothelial cells, causing the loss of antithrombotic protective factors with the influence and leakage of tissue factor, fibrinogen, von Willebrand factor, and plasminogen activator inhibitor 1 (13). In response to endothelial damage and inflammation, these prothrombotic changes promote platelet activation and fibrin formation, further amplifying complement activation (15, 17).

Thromboembolic complications are common in hospitalized adults with severe COVID-19, leading to a high level of disability and mortality (18, 19). Although much is known about such pathological conditions in adults, data regarding children are limited. The overall prevalence of thrombosis in pediatric patients with COVID-19 is significantly lower than in adults, limiting our understanding of the disease in this age group (20).

Studies have identified risk factors for thromboembolism in children, including older age (≥ 12 years), thrombophilia or venous thromboembolism in the medical history, burns, active oncologic or hematologic disease, signs of venous congestion or heart failure, nephrotic syndrome, estrogen therapy, active systemic infection, obesity, diabetes, severe dehydration, recent surgery or trauma, autoimmune diseases, antiphospholipid syndrome, sickle cell anemia, prolonged patient immobilization (non-invasive or invasive lung ventilation), and the presence of intravenous catheters (20–22). These factors, combined with the severity of the illness, mechanical ventilation, and elevated D-dimer levels, can be used for risk stratification and the prescription of heparin thromboprophylaxis (21).

Thus, in the era of COVID-19, substantial knowledge exists regarding severe complications such as thromboembolism in adults, but data regarding children are limited. The presence of coagulopathy might be regarded as a factor contributing to the severity of COVID-19 and its associated mortality. This observation could assist healthcare professionals in recognizing cases with unfavorable outcomes among COVID-19 patients (23, 24). The overall prevalence of thrombosis in children is noticeably lower than in older individuals, restricting our understanding of the role of thromboinflammation in the course of COVID-19 in this age group. The role of coagulation abnormalities in the development of long COVID is not fully understood at this time. Therefore, further research and heightened vigilance among healthcare professionals are necessary concerning these pathological conditions.

Moreover, persistent endotheliopathy and heightened coagulability frequently occur in individuals recovering from COVID-19, suggesting the intriguing possibility that these factors could play a role in the development of long COVID (2, 25).

The aim of our study was to investigate the coagulation profile in patients with COVID-19 based on age and their role in the development of long COVID.

## Materials and methods

### Study design and participants

We conducted a prospective single-center cohort study from September 2022 to August 2023. The study involved 190 patients younger than 18 years who were hospitalized at the Ternopil City Children’s Hospital, Ukraine due to COVID-19. SARS-CoV-2 infection was confirmed by polymerase chain reaction (PCR) (nasal swab) or rapid tests or positive serological analysis (IgM). The study was performed following the 1975 Declaration of Helsinki (as revised in 2000) and approved by the I. Horbachevsky Ternopil National Medical University Ethics Committee (Minutes № 70 from August 1, 2022). Written informed consent was obtained from all participants (children and their parents) in the study.

All patients were divided into two groups: less than 6 years and over 6 years. Baseline and clinical characteristics were obtained from all patients. Baseline characteristics included the age and gender of the patients, while clinical features encompassed the symptoms of COVID-19, the presence of comorbid conditions, and disease severity. Upon admission to the hospital, children also underwent a complete blood count (CBC), and the level of C-reactive protein (CRP) was determined. Additionally, a coagulation profile, which included prothrombin time (PT), activated partial thromboplastin time (aPTT), and fibrinogen level, was worked out. To determine the coagulation status, we also measured the level of D-dimer. The reference range for PT was considered as 12-15 seconds, for aPTT – 25-35 seconds, for fibrinogen – 2-4 g/L, for D-dimer – less than 250 ng/ml. Lymphopenia was defined as lymphocyte levels less than 2x10^9^/L, elevated CRP as their levels were over 5 mg/L.

After discharge from the hospital, patients were monitored for the presence of long COVID symptoms. Surveys regarding the presence of symptoms were conducted using the International Severe Acute Respiratory and Emerging Infection Consortium (ISARIC)/IP4C Global Pediatric Covid-19 follow-up Case report form at 1, 3, 6 months, and 1 year after discharge from the hospital. Participation in the survey and subsequent examination was voluntary. In children up to 6 years old, the survey questions were answered by parents. The presence of ‘Long COVID’ was determined according to WHO criteria, and was defined as continuation or development of new symptoms 3 months after the initial SARS-CoV-2 infection, with these symptoms lasting for at least 2 months without other explanation (26).

Attention was also given to the presence of comorbid conditions. Comorbidities included diseases of the nervous system; diseases of the digestive system; heart diseases; respiratory diseases (excluding asthma); asthma; allergic rhinitis/hay fever; atopic dermatitis/eczema; food allergy; renal/kidney problems; overweight or obesity; undernutrition; diabetes; other endocrine diseases (not diabetes); rheumatologic diseases. Overweight and obesity of children over 2 years was defined according to the AAP Clinical Practice Guideline for the evaluation and treatment of children and adolescents with obesity (27). Overweight was defined as a body mass index (BMI) at or above the 85th percentile and below the 95th percentile, and obesity was defined as a BMI at or above the 95th percentile. Overweight and obesity in children less than 2 years of age were determined based on WHO criteria: overweight was identified when weight-for-height exceeded 2 standard deviations, and obesity was identified when weight-for-height exceeded 3 standard deviations above WHO Child Growth Standards median. Undernutrition in children under 2 years of age was defined when weight-for-height was below 2 standard deviation according to WHO Child Growth Standards median, and in children over 2 years of age when BMI was below 2 standard deviation according to CDC Growth calculator for 2 to 20 years. Children with hematological diseases; tuberculosis; malignancies were excluded from the study. The severity of COVID-19 was determined using the National Institutes of Health COVID-19 Treatment Guidelines (28).

**Figure 1:**
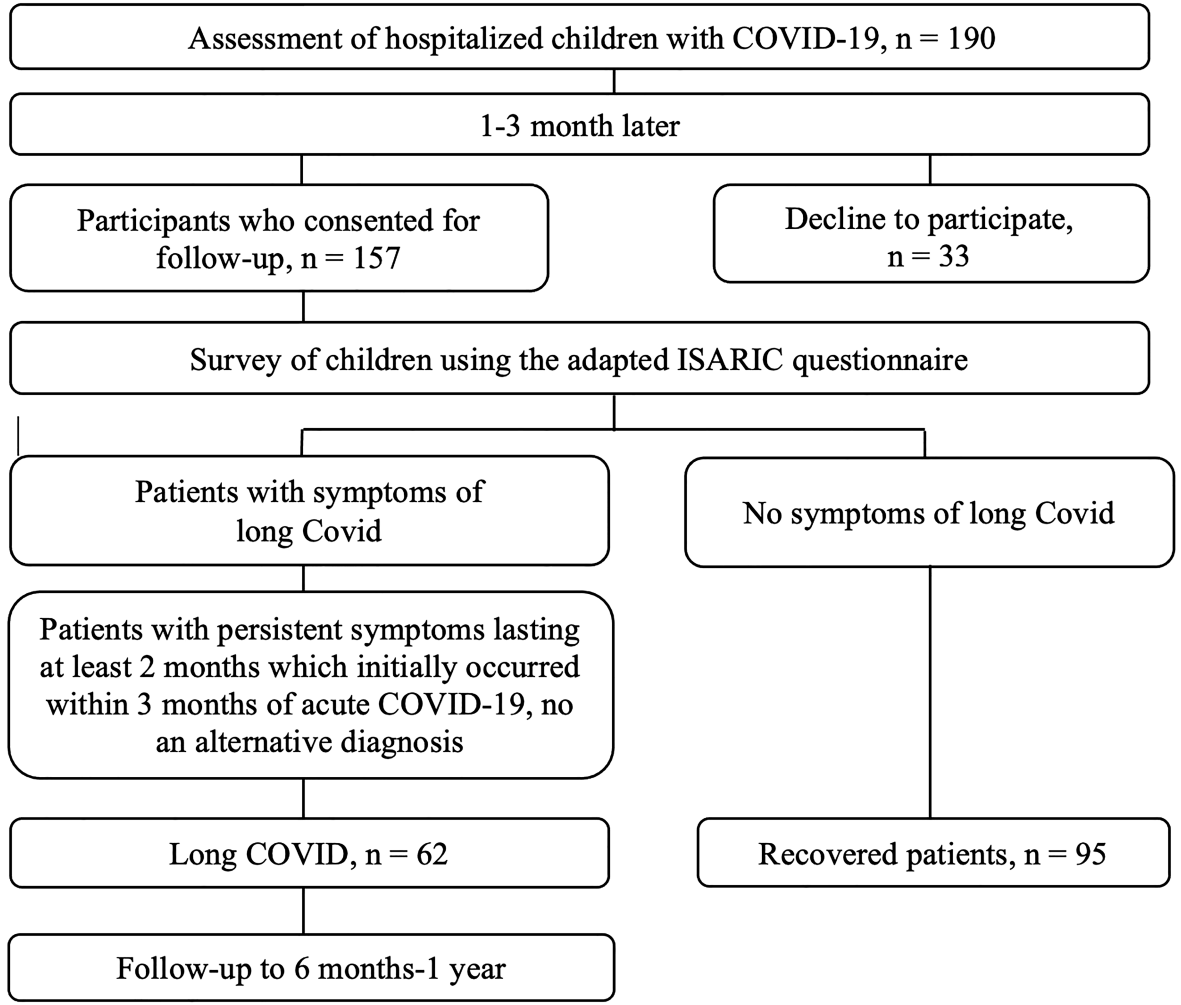
Patients flow diagram.

Among 190 patients with COVID-19, 157 respondents (82.6%) agreed to further surveys and necessary examinations, while the remaining 33 respondents declined to participate in the survey and examination. Among the 157 respondents, 62 patients (39.5%) had long COVID symptoms according to the WHO definition, while the rest (95 patients) did not have symptoms of long COVID (fully recovered). Children classified as fully recovered were those who reported the absence of lingering symptoms during the follow-up period after the onset of acute COVID-19 symptoms at least 8 weeks. Patients flow diagram is presented in **Figure 1**.

### Statistical analysis

Statistical analysis was performed using STATISTICA 10. Qualitative variables are shown as absolute frequencies and percentages. Quantitative variables were tested using Kolmogorov**–**Smirnov test or histogram for normal distribution and are expressed as median and interquartile range (IQR), when appropriate. For quantitative variables the Mann–Whitney test was performed. For categorical data chi-squared and chi-squared with Yates correction tests were used. P-values of <0.05 were considered as statistically significant.

Odds ratio (OR) and 95% confidence intervals was determined to explore the influence of potential risk factors on the development of long COVID. For this purpose we used only features that were statistically significant.

## Results

### Characteristics of hospitalized children with COVID-19

The study included 190 hospitalized children with COVID-19. The median age was 1.35 years, with 147 (77.4%) patients being less than 6 years old and 43 (22.6%) over 6 years old. Baseline and clinical characteristics of the patients with COVID-19 are presented in **Table 1**. Boys slightly outnumbered girls in the patient cohort (54.2%), and this trend was observed in both age groups.

**Table 1:** Baseline and clinical characteristics of the patients with COVID-19.

Characteristic	Total	Under 6 years	Over 6 years	P
	190	147	43	
	Median (interquartile range, IQR) or n (%)			
Age, years	1.35 (0.65; 5.5)	0.9 (0.5; 1.8)	10.2 (7.0; 14.5)	
Female	87 (45.8)	68 (46.3)	19 (44.2)	0.8104
Symptoms: fever respiratory gastrointestinal profound fatigue appetite loss others	178 (93.7) 142 (74.7) 54 (28.4) 103 (54.2) 81 (42.6) 35 (18.4)	139 (94.6) 108 (73.5) 42 (28.6) 81 (55.1) 66 (44.9) 23 (15.6)	39 (90.7) 34 (79.1) 12 (28.0) 22 (51.2) 15 (34.9) 12 (27.9)	0.3600 0.4572 0.9323 0.6484 0.2428 0.0681
Comorbidities: allergic pathology nutritional disorders overweight obesity undernutrition heart diseases nervous system diseases kidney problems digestive system diseases thyroid diseases	63 (33.2) 45 (23.7) 44 (23.2) 20 (10.5) 11 (5.8) 13 (6.8) 10 (5.2) 17 (8.9) 6 (3.2) 5 (2.6) 2 (1.1)	45 (30.6) 29 (19.7) 24 (16.3) 9 (6.1) 8 (5.4) 5 (3.4) 6 (4.1) 11 (7.5) 2 (1.4) 1 (0.7) 0 (0)	18 (41.9) 16 (37.2) 20 (46.5) 11 (25.6) 3 (7.0) 8 (18.6) 4 (9.3) 6 (14.0) 4 (9.3) 4 (9.3) 2 (4.7)	0.1682 **0.0106** **0.0001** **0.0003** 0.7047 **0.0005** 0.1775 0.1910 **0.0337** **0.0103** 0.0752
Severity mild/moderate severe/critical	179 (94.2) 11 (5.8)	140 (95.2) 7 (4.8)	39 (90.7) 4 (9.3)	0.2621 0.2621
Neutrophils, 10^9^/L	2.36 (1.17; 3.42)	2.15 (1.23; 3.46)	2.99 (1.87; 5.32)	0.0546
Lymphocytes, 10^9^/L Lymphopenia	2.25 (1.19; 3.99) 78 (41.1)	2.51 (1.47; 4.63) 47 (32.0)	1.2 (0.81; 2.09) 31 (72.1)	**<0.0001** **<0.0001**
CRP, mg/L Elevated CRP	5.48 (1.41; 14.5) 89/169 (52.7)	5.32 (1.41; 14.5) 68/131 (51.9)	6.27 (1.09; 16.92) 21/38 (55,3)	0.6995 0.7481

CRP, C-reactive protein; Significant indicators are highlighted in bold.

Among the symptoms of COVID-19, fever was most commonly observed (93.7%), followed by respiratory symptoms (74.7%) and general symptoms, with profound fatigue (54.2%) and appetite loss (42.6%) being prominent. Respiratory symptoms were dominated by runny nose (113, 59.5%), cough (88, 46.3%), hoarseness (30, 15.8%), and sore throat (13, 6.8%). Gastrointestinal symptoms were less common (28.4%) and included vomiting, diarrhea, and abdominal pain. Other symptoms included headache, arthralgia, myalgia, rashes, drowsiness, dizziness, and urinary disturbances. There was no significant difference in the frequency of symptoms between patients with COVID-19 under 6 years old and those over 6 years old.

Comorbid conditions were present in a third of the patients (33.2%), and 16 (8.4%) of them had two or more comorbidities. Allergic pathology (23.7%) predominated among comorbid conditions, and was presented by food allergy, atopic dermatitis, allergic rhinitis, and asthma. Nutritional disorders occurred in 23.2%: overweight in 20 (10.5%), obesity in 11 (5.8%), and undernutrition in 13 (6.8%). Diseases of the nervous system (epilepsy or history of seizure, encephalopathy, infantile cerebral palsy, developmental delay) were observed in 8.9% of patients. Heart diseases (congenital heart diseases, arrhythmia, arterial hypertension) occurred less frequently - in 5.2%; kidney problems (urinary tract infections, chronic kidney disease) - in 3.2%; diseases of the digestive system (functional dyspepsia, colitis, duodenal atresia, dolichocolon) - in 2.6% of patients. Thyroid diseases were observed in 2 (1.1%) patients. There were no children with diabetes, rheumatologic diseases among those surveyed. Although comorbid conditions were generally 1.4 times more common in children over 6 years old, this difference was not statistically significant. However, allergic pathology, diseases of the digestive system, and kidney problems were significantly more common in children over 6 years of age (p=0.0106, p=0103, and p=0.0337, respectively).

Children mostly had a mild/moderate course of COVID-19 (94.2%), with no significant difference between groups. The severity of the condition in patients was related to severe respiratory disease. There were 17 (8.9%) patients with COVID-19 related pneumonia. Eleven patients were in intensive care units and three of them required short-term ventilation. Thrombosis was not recorded in any case. Seven patients were on oxygen therapy.

The median neutrophil level was 2.36 x 10^9/L (IQR: 1.17; 3.42), and lymphocytes - 2.25 x 10^9/L (IQR: 1.19; 3.99). The difference in neutrophil levels between patient groups under 6 years and over 6 years was not significant, while the lymphocyte level was likely lower in the group of patients with COVID-19 over 6 years. Lymphopenia was observed more than twice as often in patients older than 6 years, and the difference between groups was statistically significant (p<0.0001). However, the CRP level, although slightly higher in the group of patients over 6 years, was not significantly different (**Table 1**).

### Platelets and coagulation profile of children with COVID-19

In the majority of children with COVID-19 upon admission, the platelet count was within the normal range (86.8%) and ranged from 95 to 681 x 10^9/L, and it was significantly higher in children under 6 years, p=0.0310 (**Table 2**). Platelet count decrease (6.3%) and increase (6.9%) were observed in almost equal proportions, with no significant difference based on age.

**Table 2:** Platelets and coagulation profile of children with COVID-19.

Parameter	Total	Under 6 years	Over 6 years	P
	174	137	37	
	Median (interquartile range, IQR) or n (%)			
Thrombocytes, 10^9^/L < 150 10^9^/L > 400 10^9^/L	247 (207; 310) 11/174 (6.3) 12/174 (6.9)	255 (208; 321) 7/137 (5.1) 11/137 (8.0)	216 (200; 280) 4/37 (10.8) 1/37 (2.7)	**0.0310** 0.2060 0.2566
PT, sec > 15 sec < 12 sec	14.7 (13.5; 15.7) 66/166 (39.8) 6/166 (3.6)	14.4 (13.4; 15.5) 44/129 (34.1) 5/129 (3.9)	15.3 (14.6; 16.4) 22/37 (59.5) 1/37 (2.7)	**0.0089** **0.0055** 0.7361
aPTT, sec > 35 sec < 25 sec	38.1 (34.1; 44.6) 114/165 (69.1) 1/165 (0.6)	39.2 (34.8; 45.8) 94/128 (73.4) 0/128	35.4 (31.4; 40.0) 20/37 (54.1) 1/37 (2.7)	**0.0132** **0.0246** 0.0621
Fibrinogen, g/L > 4 g/L < 2 g/L	2.29 (1.72; 3.09) 12/165 (7.3) 64/165 (38.8)	2.08 (1.63; 2.94) 6/128 (4.7) 57/128 (44.5)	3.04 (2.5; 3.5) 4/37 (10.8) 7/37 (18.9)	**0.0006** 0.1692 **0.0049**
Ferritin, ng/mL (normal 22-350)	46.8 (24.8; 88.2)	46.1 (25.4; 102.7)	55.9 (17.4; 61.9)	0.4262
D-dimer, ng/mL > 250 ng/mL	239 (100; 560) 36/80 (45.0)	241 (100; 560) 27/59 (45.8)	214 (100; 750) 9/21 (42.9)	**0.0471** 0.8182

PT, prothrombin time; aPTT, activated partial thromboplastin time; Significant indicators are highlighted in bold.

Only in 9/165 (5.5%) hospitalized COVID-19 patients, all coagulation profile parameters assessed in this study were within normal limits. PT upon admission in children with COVID-19 ranged from 10.6 to 20.7 seconds, with a median PT of 14.7 seconds, and in 39.8% of examined patients upon admission, it was more than 15 seconds. Children over 6 years old had a significantly higher median PT value (p= 0.0089), and in a larger number of children, it was more than 15 seconds (p= 0.0055). Activated partial thromboplastin time (aPTT) ranged from 21.7 to 79.4 seconds, with a median of 38.1 seconds, and a higher median aPTT was observed in the group of patients under 6 years (p=0.0132). Increased aPTT above the reference value (35 seconds) was observed in 69.1% of patients, with a predominance in the group of children under 6 years (p=0.0246). Fibrinogen levels ranged from 0.62 to 5.85 g/L. Higher fibrinogen levels were observed in patients with COVID-19 over 6 years (p=0.0006). Although the percentage of children over 6 years with a fibrinogen level exceeding 4 g/L was more than twice that of children under 6 years, the difference was not statistically significant. However, a significant number of children (38.8%) had a fibrinogen level less than 2 g/L, and in this cohort, significantly more children were under 6 years old (p=0.0049). Ferritin levels ranged from 3.8 to 440 ng/mL, with a more frequent observation of reduced levels (11/56), and only in 2 cases, it was elevated. We did not find a significant difference in the median ferritin level based on age.

D-dimer levels ranged from 10 to 10,000 ng/mL. Elevated D-dimer levels were observed in 45% of children upon admission to the hospital. Children under 6 years had a significantly higher median of D-dimer than patients over 6 years (p=0.0471), but high levels (more than 250 ng/mL) were observed equally often in both age groups (p=0.8182).

All patients with COVID-19 related pneumonia had changes in the coagulation profile. Changes in PT were observed in 6/17 (35.3%) patients, and fibrinogen changes were seen in 2/17 (11%) patients with COVID-19 related pneumonia, without significant difference from the rest of the hospitalized patients. Although the proportion of children with an elevated aPTT was higher in children with pneumonia compared to those without diagnosed pneumonia (82.4% versus 67.6%, respectively), although the difference was not significant (p=0.2115). In all hospitalized patients with COVID-19 related pneumonia, the level of D-dimer was elevated, which was statistically significant compared to patients without pneumonia (100% versus 38.9%, respectively; p=0.0010). The median D-dimer level was also likely higher in patients with pneumonia (932 ng/mL; IQR: 460.5; 1195 versus 239 ng/mL; IQR: 100; 560, respectively; p=0.0054).

Antithrombotic prophylaxis was administered to 5 (2.6%) patients with critical COVID-19 related pneumonia who were admitted to the intensive care units. Low-dose unfractionated heparin was used.

### Clinical characteristics and coagulation profile of children with long COVID

Among the hospitalized 190 patients with COVID-19, 157 patients or their parents consented to further examination: 120 children under 6 years and 37 children over 6 years. Among them, signs of long COVID were found in 62 children (39.5%): 43 (35.8%) were children under 6 years and 19 (51.4%) were children over 6 years. The patients were followed for a period from 6 months to 1 year, with a mean of 9.5 months. Baseline and clinical characteristics of patients with long COVID and without symptoms of long COVID are presented in **Table 3**. The median age of patients with long COVID was 1.9 (IQR: 1.17; 3.42) years and was significantly higher than in patients without symptoms of long COVID (p=0.0181). Among patients with long COVID, females predominated (58.1% versus 40%, p=0.0267), with a more pronounced difference in the group over 6 years (68.4% versus 22.2%, p=0.0048).

**Table 3:** Baseline and clinical characteristics of patients with long COVID-19 and recovered.

Parameter	Long COVID			Recovered			P1	P2	P3
	Total	Under 6 years	Over 6 years	Total	Under 6 years	Over 6 years	Total	Under 6 years	Over 6 years
	62	43	19	95	77	18			
	Median (interquartile range, IQR) or n (%)								
Age, years	1.9 (0.8; 7.9)	1.3 (0.6; 1.9)	12.9 (8.3; 14.5)	1.0 (0.6; 4.6)	0.9 (0.5; 1.7)	9.4 (6.9; 14.8)	**0.0181**	0.1220	0.3462
Female	36 (58.1)	23 (53.5)	13 (68.4)	38 (40.0)	34 (44.2)	4 (22.2)	**0.0267**	0.3263	**0.0048**
Comorbidities: allergic pathology nutritional disorders overweight obesity undernutrition heart diseases nervous system diseases kidney problems digestive system diseases thyroid diseases	30 (48.4) 27 (43.5) 24 (38.7) 9 (14.5) 8 (12.9) 7 (11.3) 5 (8.1) 8 (12.9) 4 (6.5) 3 (4.8) 1 (1.6)	18 (41.9) 16 (37.2) 10 (23.3) 2 (4.7) 5 (11.6) 3 (7.0) 0 (0) 3 (7.0) 2 (4.7) 0 (0) 0 (0)	12 (63.2) 11 (57.9) 14 (73.7) 7 (36.8) 3 (15.8) 4 (21.1) 5 (26.3) 5 (26.3) 2 (10.5) 3 (15.8) 1 (5.3)	26 (27.4) 16 (16.8) 19 (20.0) 11 (11.6) 3 (3.2) 5 (5.3) 4 (4.2) 7 (7.4) 2 (2.1) 2 (2.1) 1 (1.1)	20 (26.0) 11 (14.3) 13 (16.9) 7 (9.1) 3 (3.9) 3 (3.9) 4 (5.2) 6 (7.8) 0 (0) 1 (1.3) 0 (0)	6 (33.3) 5 (27.8) 6 (33.3) 4 (22.2) 0 (0) 2 (11.1) 0 (0) 1 (5.6) 2 (11.1) 1 (5.6) 1 (5.6)	**0.0072** **0.0002** **0.0102** 0.5895 **0.0194** 0.1647 0.3099 0.2488 0.1650 0.3403 0.7596	0.0728 **0.0039** 0.3951 0.3759 0.1035 0.4578 0.1285 0.8708 0.0563 0.4530 na	0.0697 0.0646 **0.0138** 0.3308 0.0786 0.4122 **0.0193** 0.2379 0.9543 0.3163 0.9686
COVID-19 severity mild/moderate severe/critical	57 (91.9) 5 (8.1)	41 (95.3) 2 (4.7)	16 (84.2) 3 (15.8)	90 (94.7) 5 (5.3)	73 (94.8) 4 (5.2)	17 (94.4) 1 (5.6)	0.4823 0.4823	0.8958 0.8958	0.3163 0.3163
Neutrophils, 10^9^/L	2.57 (1.63; 3.3)	2.19 (1.39; 3.28)	3.05 (2.41; 3.52)	2.58 (1.33; 4.53)	2.21 (1.19; 4.08)	3.69 (1.68; 6.04)	0.9617	0.9497	0.5499
Lymphocytes, 10^9^/L Lymphopenia	1.98 (1.03; 3.19) 25/53 (47.2)	2.54 (1.33; 4.7) 12/37 (32.4)	1.13 (0.77; 1.87) 13/16 (81.3)	2.29 (1.36; 4.05) 36/89 (40.5)	2.52 (1.66;4.46) 24/72 (33.3)	1.25 (0.79; 2.15) 12/17 (70.6)	0.9617 0.4339	0.4875 0.9246	0.8712 0.4751
CRP, mg/L Elevated CRP	3.82 (1,09; 9,72) 21/53 (39.6)	3.48 (1.22; 9.6) 13/36 (36.1)	4.2 (1.09; 17.19) 8/17 (47.1)	8.17 (1.8; 16.1) 49/83 (59.0)	8.58 (2,21; 16.0) 40/68 (58.8)	6.54 (0.79; 23.6) 9/15 (60.0)	**0.0444** ** 0.0272**	**0.0425** ** 0.0275**	0.5839 0.4641

CRP, C-reactive protein; Significant indicators are highlighted in bold.

**Figure 2:**
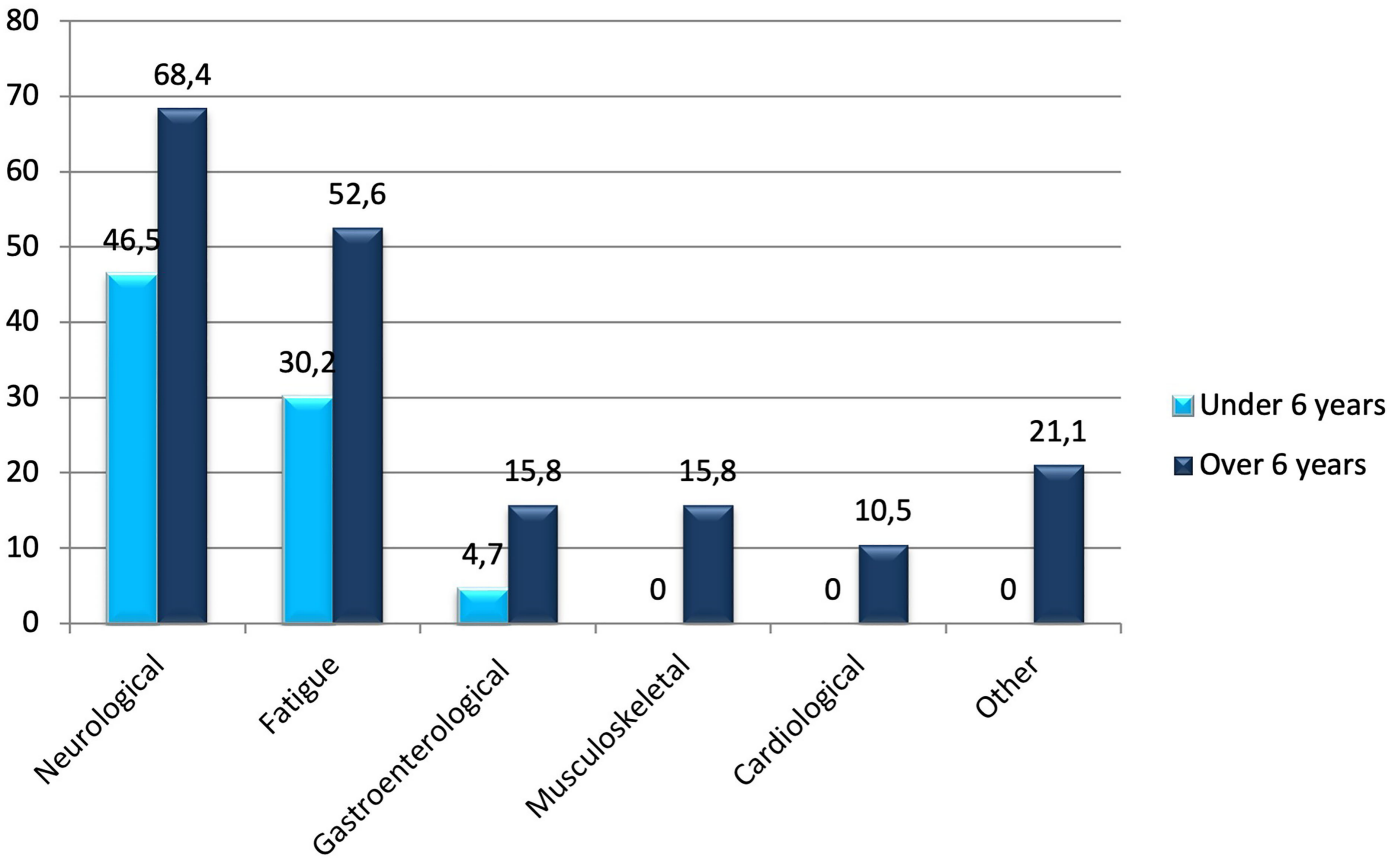
Symptoms (%) of long COVID based on age.

In the clinical presentation of children experiencing long COVID, the most prevalent symptoms were fatigue (23; 37.1%) and neurological manifestations (33; 53.2%) such as headache, poor concentration, sleep disorders, memory decline, and irritability. Gastroenterological symptoms, including nausea, abdominal pain, and increased level of liver enzymes were less common (5; 8.1%). Respiratory symptoms, such as cough, dyspnea, and sore throat, occurred in 4 patients (6.5%), while musculoskeletal symptoms, including myalgia and arthralgia, were observed in 3 patients (4.8%). Cardiological symptoms, such as postural tachycardia syndrome and tachycardia, were reported by 2 patients (3.2%). Other reported symptoms comprised rashes (2; 3.2%) and low-grade fever (2; 3.2%). Symptoms of long COVID based on age are presented in **Figure 2**.

Comorbidities were more frequently found in patients with long COVID (p=0.0072). Among comorbid conditions in patients with long COVID, allergic pathology (p=0.0002), nutritional disorders (p=0.0102), including obesity (p=0.0194), and heart diseases in the group of patients over 6 years old (p=0.0193) were significantly more common (**Table 3**).

Children with mild/moderate severity of COVID-19 predominated (91.9%) among patients with long COVID, and the severity of SARS-CoV-2 infection did not affect the development of long COVID symptoms according to our data. Also, we did not observe an impact of COVID-19 related pneumonia on the development of long COVID symptoms. Pneumonia was observed in 7 out of 62 patients who developed long COVID (11.3%), while among 95 patients who did not have symptoms of long COVID, pneumonia occurred in 9 children (9.5%). And although patients who were in the intensive care unit developed symptoms of long COVID twice as often, the difference was not significant (6/62; 9.7% versus 5/95; 5.3%, respectively; p=0.2763). There was no difference in the median absolute values of neutrophils and lymphocytes between patients who developed symptoms of long COVID and those without symptoms of long COVID. However, in patients who did not develop symptoms of long COVID, significantly higher median CRP values were observed (8.17 mg/L versus 3.82 mg/L, p=0.0444), and the same pattern was observed in the age group under 6 years (8.58 mg/L versus 3.48 mg/L, p=0.0425). Elevated CRP levels were more frequently observed in patients who did not develop long COVID symptoms later (59% versus 39.6%, p=0.0272), and a similar pattern was observed in patients under 6 years (p=0.0275).

The platelet levels and coagulation profile in patients with symptoms of long COVID and recovered is presented in **Table 4**. As seen in **Table 4**, there was no significant difference between the median of platelet levels and coagulation profile indicators in patients with COVID-19 who developed long COVID symptoms and those without long COVID symptoms. Only a tendency towards an increase in the median aPTT was observed in patients under 6 years with long COVID (40.1 sec versus 38.1 sec, p= 0.0832). And although there was no significant difference between the groups with long COVID and recovered in the percentage of patients with prolonged PT, aPTT, elevated levels of fibrinogen, and D-dimer, the percentage of children with abnormal PT was higher in the long COVID group (53% versus 36.1%, p=0.0432).

**Table 4:** Platelets and coagulation profile in patients with long COVID and recovered.

Parameter	Long COVID			Recovered			P1	P2	P3
	Total	Under 6 years	Over 6 years	Total	Under 6 years	Over 6 years	Total	Under 6 years	Over 6 years
	62	43	19	95	77	18			
	Median (interquartile range, IQR) or n (%)								
Thrombocytes, 10^9^/L < 150 10^9^/L > 400 10^9^/L Abnormal	241.0 (214.5; 281.5) 3/56 (5.4) 3/56 (5.4) 6/56 (10.7)	242.5 (214.5; 280.0) 2/40 (5/0) 2/40 (5/0) 4/40 (10)	236.0 (211.5; 288.0) 1/16 (6.3) 1/16 (6.3) 2/16 (12.5)	261.0 (207.0; 329.0) 6/94 (6.4) 6/94 (6.4) 12/94 (12.8)	274.5 (216.5; 336.5) 4/76 (5.3) 6/76 (7.9) 10/76 (13.2)	210.5 (202.0; 297.0) 2/18 (11.1) 0/18 (0) 2/18 (11.1)	0.1556 0.7980 0.7980 0.7084	0.0746 0.9515 0.5587 0.6198	0.4582 0.6179 0.2179 0.9002
PT, sec > 15 sec < 12 sec Abnormal	14.8 (13.7; 16.0) 25/52 (48.1) 3/52 (5.8) 28/52 (53.8)	14.7 (13.4; 15.5) 15/38 (39.5) 1/38 (2.6) 16/38 (42.1)	15.6 (14.8; 17.8) 10/14 (71.4) 2/14 (14.3) 12/14 (85.7)	14.5 (13.4; 15.6) 29/83 (35.0) 1/83 (1.2) 30/83(36.1)	14.3 (13.3; 15.3) 20/66 (30.3) 1/66 (1.5) 21/66 (31.8)	15.3 (14.6; 15.9) 9/17 (52,9) 0/17 (0) 9/17 (52.9)	0.1959 0.1295 0.1280 **0.0432**	0.3802 0.3406 0.6897 0.2913	0.3308 0.2930 0.0977 0.0521
aPTT, sec > 35 sec < 25 sec Abnormal	39.5 (34.6; 47.6) 38/52 (73.1) 2/52 (3.9) 40/52 (77)	40.1 (35.4; 47.9) 29/38 (76.3) 1/38 (2.6) 30/38 (78.9)	36.9 (30.5; 42.4) 9/14 (64.3) 1/14 (7.1) 10/14 (71.4)	37.7 (33.3; 41.4) 55/82 (67.1) 0/82 (0) 55/82 (67.1)	38.1 (33.4; 42.9) 44/65 (67.7) 0/65 (0) 44/65 (67.7)	36.0 (33.3; 40.0) 11/17 (64.7) 0/17 (0) 11/17 (64.7)	0.1348 0.4624 0.0736 0.2213	0.0832 0.3527 0.1888 0.2204	0.9525 0.9806 0.2626 0.6903
Fibrinogen, g/L > 4 g/L < 2 g/L Abnormal	2.15 (1.63; 3.03) 2/52 (3.9) 21/52 (40.4) 23/52 (44.3)	2.04 (1.56; 2.70) 1/38 (2.6) 18/38 (47.4) 19/38 (50)	2.97 (2.07; 3.55) 1/14 (7.1) 3/14 (21.4) 4/14 (28.5)	2.53 (1.86; 3.22) 9/81 (11.1) 28/81 (34.6) 37/81 (45.7)	2.26 (1.77; 2.98) 6/64 (9.4) 26/64 (40.6) 32/64 (50)	3.15 (2.80; 3.54) 3/17 (17.7) 2/17 (11.8) 5/17 (29.5)	0.1789 0.1377 0.4974 0.8699	0.1968 0.1928 0.5061 1.0000	0.4273 0.3853 0.4666 0.9591
Ferritin, ng/mL (normal 22-350)	55.9 (26.4; 86.9)	50.9 (26.4; 102.7)	56.1 (4.0; 59.0)	46.1 (22.7; 86.7)	46.1 (25.4; 86.5)	52.1 (16.2; 158.1)	0.8159	0.5880	0.4555
D-dimer, ng/mL > 250 ng/mL	239 (100; 840) 9/23 (39.1)	240 (100; 920) 6/16 (37.5)	176 (102; 750) 3/7 (42.9)	239 (100; 605) 20/44 (45.5)	272 (100; 605) 16/32 (50.0)	169 (48; 695) 4/12 (33.3)	0.8019 0.6199	0.8956 0.3541	0.6421 0.6780

PT, prothrombin time; aPTT, activated partial thromboplastin time; Significant indicator is highlighted in bold.

We also determined the OR to identify potential risk factors for the development of long COVID. To achieve this, we utilized parameters that were statistically significant according to the data in **Tables 3**, **4**. The results are presented in **Table 5**. All indicators, except for the presence of heart diseases, were statistically significant, including abnormal PT (p=0.0445).

**Table 5:** Risk factors associated with long COVID development.

Parameter	Long Covid	Recovered	OR	95%	P
	n, %	n, %			
Age over 6 years	19/62 (30.6)	18/95 (18.9)	0.5290	0.2512-1.1143	0.0939
Female	36/62 (58.1)	38/95 (40.0)	2.0769	1.0839 – 3.9798	**0.0276**
Comorbidities allergic pathology nutritional disorders obesity heart diseases (for patients > 6 years)	30/62 (48.4) 27 (43.5) 24 (38.7) 8 (12.9) 5/19 (26.3)	26/95 (27.4) 16 (16.8) 19 (20.0) 3 (3.2) 0/18 (0)	2.4880 3.8089 2.5263 4.5432 14.0345	1.2706 – 4.8717 1.8257 – 7.9464 1.2335 – 5.1739 1.1558 – 17.8578 0.7160 – 275.098	**0.0078** **0.0004** **0.0113** **0.0302** 0.0819
Elevated CRP	21/53 (39.6)	49/83 (59.0)	0.4554	0.2254 – 0.9198	**0.0283**
Abnormal PT	28/52 (53.8)	30/83(36.1)	2.0611	1.0179 – 4.1737	**0.0445**

OR, Odds ratio; CRP, C-reactive protein; PT, prothrombin time; Significant indicators are highlighted in bold.

## Discussion

The pathological aspects of thromboinflammation involve interconnected processes, including endothelial damage, thrombus formation through platelet activation, and the coagulation cascade (14, 16, 19). In COVID-19, thromboinflammation induces endothelial damage by generating proinflammatory cytokines and triggering platelets and the complement system (2, 23). The study of the coagulation profile and platelet levels in patients with COVID-19 and the impact of their abnormalities on the severity of the disease, its consequences, and the duration of disorders have been a focal point for researchers throughout the COVID-19 era.

In our study, we identified coagulation abnormalities in hospitalized children with COVID-19. Specifically, prolongation of PT > 15 sec was observed in 39.8% of patients, aPTT > 35 sec in 69.1%, elevated fibrinogen levels in 7.3%, and elevated D-dimer in 45% of children. Our findings are consistent with the results of other studies that have reported abnormalities in the coagulation profile in patients with COVID-19 (25, 29, 30). However, most of these studies are related to the adult population. For instance, Jin et al. (30) noted frequent prolongation of PT and aPTT in critically ill adults with COVID-19, which had significant prognostic value when assessed during hospitalization. Researchers also indicated no significant difference in aPTT between patients with and without thrombosis (30). Noni et al. also investigated coagulation changes in pediatric patients with COVID-19 (29). They similarly found prolongation of PT in 36.3% of patients, aPTT exceeding 39 sec in 12.6%, and elevated fibrinogen levels in 8.5%, largely consistent with our results. However, they reported elevated D-dimer levels in 84.3%, nearly twice the percentage in our patients.

We did not assess the coagulation profile based on the severity of the disease since our cohort primarily consisted of patients with mild/moderate severity of COVID-19. Although other studies indicate the influence of PT and aPTT at admission on the outcomes of SARS-CoV-2 infection (31), confirmed by another study showing a sensitivity of 100% and specificity of 78.08% for PT less than 75% in predicting mortality (32). On the other hand, other research and meta-analyses have not demonstrated a correlation between the coagulation profile, including aPTT, and the severity of COVID-19 or its impact on patient mortality (24, 33, 34). At the same time, authors acknowledge the influence of markers such as PT, fibrin, and D-dimer on the progression of COVID-19, emphasizing the need for monitoring coagulation parameters to prevent the development of coagulopathies, especially in severe cases (34).

Some studies conducted among the pediatric population have shown that readily available and commonly used D-dimer, play a crucial role in predicting more severe manifestations of SARS-CoV-2 infection (35). Additionally, elevated levels of D-dimers were noted in individuals who experienced consequences, including long-term COVID-19. These findings reinforce the growing understanding of the significant involvement of coagulation and endothelial dysfunction in both acute and prolonged cases of COVID-19 in adults. This underscores the importance of identifying new biomarkers for endothelial dysfunction in children and exploring their predictive capacity for both immediate and enduring consequences (35).

In 13.2% of patients with COVID-19, changes in platelet levels were observed, with both increases and decreases occurring with equal frequency. Iba et al. (36) also note that the platelet levels in patients with SARS-CoV-2 infection are generally within the normal range, distinguishing it from disseminated intravascular coagulation (DIC) and sepsis-induced coagulopathy (SIC). Even in the presence of elevated D-dimer levels and prolonged coagulation times, COVID-19 coagulopathy differs from DIC/SIC (37).

The analysis of baseline and clinical characteristics and the coagulation profile of patients with COVID-19 based on age groups showed that patients over 6 years old had lower lymphocyte levels, which may be related to the age-specific spectrum of children. An age-related dependence of the coagulation profile was also identified. In patients under 6 years old, the median aPTT was more prolonged (p=0.0132), and the median level of D-dimer was higher (p=0.0471) compared to patients with COVID-19 over 6 years old. In this same group of patients, prolonged aPTT was observed more frequently (p=0.0246). On the other hand, the medians of PT and fibrinogen levels were higher in patients over 6 years old (p=0.0089 and p=0.0055, respectively).

To the best of our knowledge, the only study on the impact of the coagulation profile on the development of persistent symptoms after COVID-19 was conducted in Italy by Di Gennaro et al. (38). Both groups (patients with persistent symptoms and those who had recovered) exhibited abnormal coagulation profiles at follow-up, but only the percentage of patients with elevated D-dimer levels was higher in the group with symptoms of long COVID. The researchers did not find differences between the medians of PT, aPTT, fibrinogen, and other coagulation profile indicators when observing patients after recovering from COVID-19, which is consistent with our study but we focused on acute SARS-CoV-2 infection. Abnormal aPTT was observed more frequently than abnormal PT (36), aligning with the results of our study. The median D-dimer and the percentage of patients with abnormal D-dimer were significantly higher in the group with the persistence of three or more symptoms at ≥12 weeks, suggesting that chronic endothelial inflammation may play a role in the development of long COVID symptoms, as D-dimer indicates coagulation activation and contributes to thromboinflammation in COVID-19 patients (39). However, the authors emphasize the need for further research to explore the role of endothelial/platelet hyperactivation and chronic inflammation in the development of long COVID (39). Prolonged elevation of D-dimer levels over three months was also observed in another study (35) in 15% of patients who recovered from COVID-19.

Our study did not demonstrate significant changes in D-dimer levels in patients with acute SARS-CoV-2 infection who later developed long COVID symptoms compared to those who recovered. However, there was a tendency toward prolongation of the median aPTT in patients under 6 years old who subsequently developed long COVID. Differences in platelets levels were also observed in this age group. In our study, a significant difference in the percentage of abnormal PT values was found in patients with long COVID compared to those who recovered (p=0.0432). As mentioned earlier, PT, along with D-dimer, influences the progression of COVID-19, emphasizing the need for its continued monitoring, especially in severe cases (34, 40). It was also demonstrated that a prolonged PT, along with cluster infection and an elevated white blood cell count, serves as an early risk factors associated with the persistence of detectable positivity in children who have recovered from COVID-19 (41).

Certainly, the observed trends in coagulation profiles, specifically the tendency toward prolonged aPTT in children under 6 years old and the significant difference in abnormal PT values in pediatric patients with long COVID, raise intriguing questions about the potential role of coagulation abnormalities in the persistence of symptoms after acute SARS-CoV-2 infection. While our study did not reveal substantial changes in D-dimer levels, the association with altered coagulation parameters in a subset of patients, especially in the younger age group, warrants further investigation.

The interplay between the coagulation system and the immune response is a complex and dynamic process. Understanding how these interactions contribute to the development and persistence of long COVID symptoms, particularly in pediatric populations, could shed light on the pathophysiological mechanisms at play. Longitudinal studies tracking coagulation profiles over time, alongside clinical symptomatology, may provide valuable insights into the evolving nature of the disease.

In a recently published study by Zanini et al., the proposal is made to characterize vascular long COVID as a potential complication of SARS-CoV-2 infection (42). This suggestion aims to streamline the diagnostic process, considering that dysregulated immune responses and pro-coagulant conditions associated with the infection can directly lead to thromboembolic complications in both arterial and venous systems.

In our study, in addition to abnormal PT, other factors influencing the development of long COVID were identified, including older age in children, female gender, and comorbid conditions such as allergic pathology and nutritional disorders, including obesity. Conversely, an elevated level of CRP during COVID-19 was a predictor of recovery. Our findings align with a systematic review supported by a meta-analysis, indicating that female gender; certain medical comorbidities such as pulmonary disease, diabetes, obesity, and organ transplantation were potential risk factors for long COVID-19 (43, 44). Another meta-analysis on risk factors for long COVID in children also confirms that older age, female gender, poor physical or mental health, severe infection, or more symptoms were predictors of long COVID (45). However, another study demonstrates that predictors of long COVID development included increased leukocytes, monocytes, neutrophils, platelets, and D-dimer (46). In this study, the CRP level was higher in patients with prolonged symptoms, although the difference was not statistically significant. Besides female gender, a high number of symptoms during acute SARS-CoV-2 infection, and intensive care unit hospitalization are noted as predictors of long COVID (47). Some authors mention an elevated CRP level as a risk factor for long COVID, but confirming data mainly relate to multisystem inflammatory syndrome in children (48). The differences also lie in the design of studies investigating risk factors for long COVID. While some researchers examine the impact of indicators during acute infection, others observe patients after recovering from the infection. A higher CRP level is more frequently observed after the acute process in cases of long COVID (49).

The impact of a lower CRP level on the development of long COVID may be explained by low-grade inflammation and its role in the progression of long COVID symptoms (49). Unique immune response characteristics, particularly a specific T-cell response to SARS-CoV-2 in women, are associated with the fact that they more frequently experience long COVID (50).

In men, acute inflammation more commonly occurs, which is intense, short-lived, and leads to tissue restoration. On the other hand, low-grade inflammation is persistent, subtle, resulting in associated damage, and its frequent manifestations include chronic fatigue, arthralgia, myalgia, anxiety, and depression (50)—precisely the symptoms characteristic of long COVID. SARS-CoV-2 infection can trigger the release of damage-associated molecular patterns and provoke low-grade inflammation (49, 51). Thus, it can be assumed that a lower CRP level in patients with subsequent development of long COVID is associated with low-grade inflammation, which subsequently leads to uncontrolled immune response activation with the release of cytokines, causing the emergence of long COVID symptoms (49). Our hypothesis requires further confirmation in a larger patient cohort. Further research should delve into the specific pathways and mechanisms underlying low-grade inflammation post-SARS-CoV-2 infection and its role in the persistence of symptoms. Exploring the nuances of immune responses in different demographic groups, as highlighted by gender disparities, may offer valuable insights into why certain individuals are more susceptible to long COVID.

### Strengths and limitation of the study

The strengths of this study are that the investigation of the coagulation profile in children with COVID-19 is the first in Ukraine. We also examined the impact of coagulation profile abnormalities during acute COVID-19 on the subsequent development of long COVID symptoms. Such studies in children are very limited, as most focus on coagulation profile abnormalities already present in long COVID.

The study has certain limitations. Primarily, it is a single-center study, and the number of patients exhibiting symptoms of long COVID was not substantial. Additionally, there is an absence of a control group of healthy children, although it is currently challenging to find children who have not experienced COVID-19. Furthermore, we assessed not only absolute values but also the percentage of deviations from the norm. On the other hand, the literature lacks data on age-specific characteristics of the coagulation profile. It is known that in young children, there is a physiologically lower level of coagulation proteins, which might have partially influenced the study results (41). Therefore, our findings regarding age-dependent disturbances in the coagulation profile highlight the need for broader investigations and studies on the coagulation profile in children of different age groups. Other researchers also emphasize the necessity of studying hemostasis in children based on age, which could be a crucial prerequisite for exploring thromboinflammation in pediatric patients (39). For a more detailed analysis of thromboinflammation in COVID-19 and its impact on the development of persistent symptoms, it is advisable to identify a broader spectrum of indicators, considering both markers of coagulation and endothelial dysfunction.

## Conclusion

This study revealed abnormalities in the coagulation profile in 94.5% of children with COVID-19, and these changes were found to be age-dependent, and it can be concluded that there are age-dependent variations in the coagulation profiles of pediatric patients with acute SARS-CoV-2 infection. Among children who developed persistent long COVID symptoms, there were a statistically higher percentage of abnormal prothrombin time values, with no significant differences noted in other coagulation profile indicators.

The study highlights the need for more extensive research into the coagulation profiles of pediatric populations, considering age-specific factors. This could enhance our understanding of thromboinflammation in COVID-19 and its potential contribution to the development of persistent symptoms.

## Data availability statement

The raw data supporting the conclusions of this article will be made available by the authors, without undue reservation.

## Ethics statement

The studies involving humans were approved by I. Horbachevsky Ternopil National Medical University Ethics Committee. The studies were conducted in accordance with the local legislation and institutional requirements. Written informed consent for participation in this study was provided by the participants’ legal guardians/next of kin.

## Author contributions

OB: Conceptualization, Data curation, Formal Analysis, Funding acquisition, Methodology, Project administration, Resources, Supervision, Writing – original draft, Writing – review and editing. VP: Data curation, Formal Analysis, Investigation, Software, Visualization, Writing – original draft, Writing – review and editing. TK: Data curation, Investigation, Writing – original draft, Writing – review and editing. LV: Data curation, Investigation, Writing – original draft, Writing – review and editing.
